# Evaluating the restoration of Lake Manzala after dredging using water quality parameters and zooplankton changes

**DOI:** 10.1038/s41598-025-98069-x

**Published:** 2025-05-10

**Authors:** Seliem M. El Sayed, Mahmoud H. Hegab, Moustafa S. Abdelhameed, Mohamed E. Goher

**Affiliations:** National Institute of Oceanography and Fisheries (NIOF), Cairo, Egypt

**Keywords:** Mediterranean lakes, Physicochemical characteristics, Zooplankton communities, Dredging and cleaning, Lake rehabilitation, Ecology, Environmental sciences, Limnology

## Abstract

Lake Manzala is the largest northern lake in Egypt and receives significant quantities of wastewater. This study was conducted in 2015 and 2022 (before and after dredging) to assess changes in physicochemical parameters [transparency, salinity, dissolved oxygen (DO), chemical oxygen demand (COD), biological oxygen demand (BOD), and nutrients] as well as the community composition of zooplankton in relation to the dredging process. Water quality parameters, particularly salinity, classified the different lake sites into three groups (north, middle, and south) in 2015 and two groups (north-middle and south) in 2022. The highest values for transparency, salinity, and DO were recorded in the northern sector, while the highest values for BOD, COD, and nutrients were found in the southern sector. A total of 43 zooplankton species were identified in 2015, compared to 31 species in 2022. Notably, the number of saline species increased in 2022 to 12 species, and their distribution extended into the northern and middle sectors. Additionally, principal component analysis (PCA) revealed that zooplankton species could be divided into saline and freshwater groups. The study concluded that the chemical parameters and zooplankton composition in 2022 differed significantly from those in 2015 due to dredging, which altered the lake’s ecology by increasing salinity and reducing nutrient levels, particularly in the northern and middle regions.

## Introduction

The northern coastline of Egypt features five lakes connected to the Mediterranean Sea. These lakes serve as a significant source of fish production, contributing over 50% of Egypt’s total fisheries output in the 1980s. However, between 2015 and 2020, this contribution declined to less than 11%^1^. Lakes are particularly vulnerable to rapid environmental changes resulting from various human activities, which lead to decreased water quality and increased eutrophication. This situation is evident in the Mediterranean lakes of Egypt, which receive substantial amounts of untreated industrial, domestic, and agricultural wastewater^2–6^. As a result of these human activities, these coastal lakes have suffered severe ecological damage, leading to a decline in fish production. Lake Manzala is the largest lake among the northern coastal lakes of Egypt, covering a surface area of approximately 572.41 km^2^^7,8^. It is recognized as one of the most significant wintering and nesting sites for various species of migratory birds^9^. Additionally, Lake Manzala is expected to play a vital role in mitigating the impacts of climate change. It is also essential for protecting coastal cities from storm surges and flooding. Furthermore, Lake Manzala is invaluable to the biodiversity of the Mediterranean region^10^. Moreover, it functions as a naturally occurring oxidation basin, acting as a buffer zone to prevent saline coastal water from infiltrating groundwater and agricultural fields, while also serving as a natural barrier between the drainage systems of the Mediterranean Sea and the Nile Delta^11^.

There are two main sources of water for Lake Manzala: (1) Saline water enters the lake from the Mediterranean Sea to the north through narrow channels, specifically the Al-Gamil, Ashtoum al-Gamil, and al-Sofara outlets^12^. Seawater flows into the lake through the al-Sofara outlets, located in the northwest corner, via several openings, with El-Boghdady being the most significant. (2) Freshwater sources enter the lake through numerous drains and pumping stations to the south, particularly Bahr al-Baqar, Hadous, Al-Mataria, Faraskur, and Al-Serw^6^. Untreated wastewater, laden with various pollutants—including heavy metals, pesticides, PCBs, high concentrations of nutrients, and organic matter—constitutes approximately 98% of the annual inflow to Lake Manzala. These pollutants have led to an increase in vegetation area while simultaneously decreasing water quality and fish productivity^13^. Additionally, the lake contains a substantial number of islands, which cover about 23% of its total area^3^. Since surface water is a complex mixture of soluble and insoluble chemicals, water quality encompasses all characteristics of a waterbody influenced by surrounding environmental factors. Waterbodies deteriorate to varying degrees due to the direct impact of dissolved and insoluble contaminants on surface water characteristics^14^. Overall, water pollution is a global issue that affects most waterways^15^ as a result of human activities and the ongoing, steady increase in pollution^16^.

The Egyptian government embarked on a massive project to restore the lake to its natural habitat. This initiative includes a large wastewater treatment project in Bahr El Baqar. Additionally, the lake restoration and treatment plan encompass an extensive dredging operation, which began in 2017 and is scheduled for completion in late 2022. The dredging process aims to increase lake depths in addition to removing islands and dense aquatic vegetation. Dredging is a lake restoration technique that involves the removal of surface-bottom layers containing pollutants, thereby controlling their release and nutrient bioavailability^17–19^. Sediment treatment is often necessary to enhance water quality and other ecosystem components^20^. Currently, dredging is the most widely used method for addressing pollutant-rich sediments. However, the positive and negative effects of dredging remain subjects of ongoing debate, particularly regarding its role in the ecosystem debate^22–27^. In several studies, dredging activities have been shown to cause significant changes in various components of lake ecosystems. Numerous investigations have documented the negative environmental impacts of dredging^26–33^. Among these negative impacts are increases in nitrate, phosphorus, ammonia, nitrogen, alkalinity, and conductivity following dredging^19,34^. Furthermore, the adverse consequences of dredging can lead to alterations in the structure and distribution of biota within a lake. In this context, Rehman et al.^25^ concluded that dredging had modified the water quality in Dal Lake, Kashmir, India, resulting in a dramatic shift in the zooplankton community structure. Factors such as predation and competition became influential in shaping the zooplankton community. Conversely, dredging may also serve as an effective strategy for improving lake environments by reducing internal nutrient loads, which can promote the dominance of less eutrophic zooplankton species and decrease the presence of indicator species associated with high eutrophication. Consequently, changes also occur in the composition of zooplankton communities^24^. Therefore, the current study aims to conduct a preliminary assessment of the environmental state of Lake Manzala by monitoring chemical changes and the community composition of zooplankton with the dredging process in its final stages. This study is characterized by the integration of chemical and biological assessments of water quality at different time intervals to evaluate the impact of dredging on the physicochemical parameters and zooplankton community structure of Lake Manzala, Egypt, by comparing data collected before (2015) and after (2022) the dredging process.

## Methods

### Site description

Lake Manzala is a rectangular, brackish, shallow, and turbid water basin. Lake Manzala is a rectangular, brackish, shallow, and turbid water basin. As of 2022, the lake measures approximately 43.1 km in length and has a mean width of 13.1 km, with depths ranging from less than 0.5 m to more than 2 m. The shallowest depths are found in the southern zone, while the deepest areas are located near the entrance to the Mediterranean Sea. The lake is situated between latitudes31° 07′ 03.2ʺʹ N and 31° 23′ 53.7ʺ N and longitudes 31° 47′ 45.4ʺ E and 32° 14′ 35.0ʺ E^35^**.** As previously mentioned, the lake receives saline water from the Mediterranean Sea through various outlets and fresh water (brackish water) from several drains that carry a significant amount of wastewater. Additionally, Lake Manzala is connected to the Suez Canal via a bit of exploring channel known as Al-Qabouti**.** Accoeding to Elshemy^3^, Khedr et al.^8^, and Abd Ellah^9^ this canal, referred to as the El-Raswa Canal, links the Suez Canal to an isolated pond in the northeastern part of the lake. This isolated pond serves as a treatment reservoir for the sewage from Port Said City. Lake Manzala is of great importance both nationally and globally; it contributed approximately 16.8% of Egypt’s natural fish production and 3.6% of the total fish production in 2021^8,36^. Over the past few decades, the surface area of Manzala Lake has steadily decreased from 1709 km^2^ in 1907 to 565.91 km^2^ in 2016, primarily due to illegal land reclamation and aquaculture practices. Furthermore, the overgrowth of aquatic vegetation and the proliferation of islets have reduced the open water area, which shrank from over 70% to approximately 45% of the total lake area between 1986 and 2016. However, due to extensive government efforts, the lake area increased to 572.41 km^2^ in 2020, and the open water area rose to about 75% of the total lake area^9,10^. According to Abd Ellah^35^, the depth of Lake Manzala increased significantly between 2016 and 2022 as a result of dredging operations, where the volume of water increased from 378.67 MCM in 2016 to 903.64 MCM in 2022 with about an increase of 524.94 MCM in the water volume. For example, the area with a depth of more than 2 m increased from 9.41 km^2^ in 2016 to 166.49 km^2^ in 2022.

### Sampling sites

**Fig. 1:**
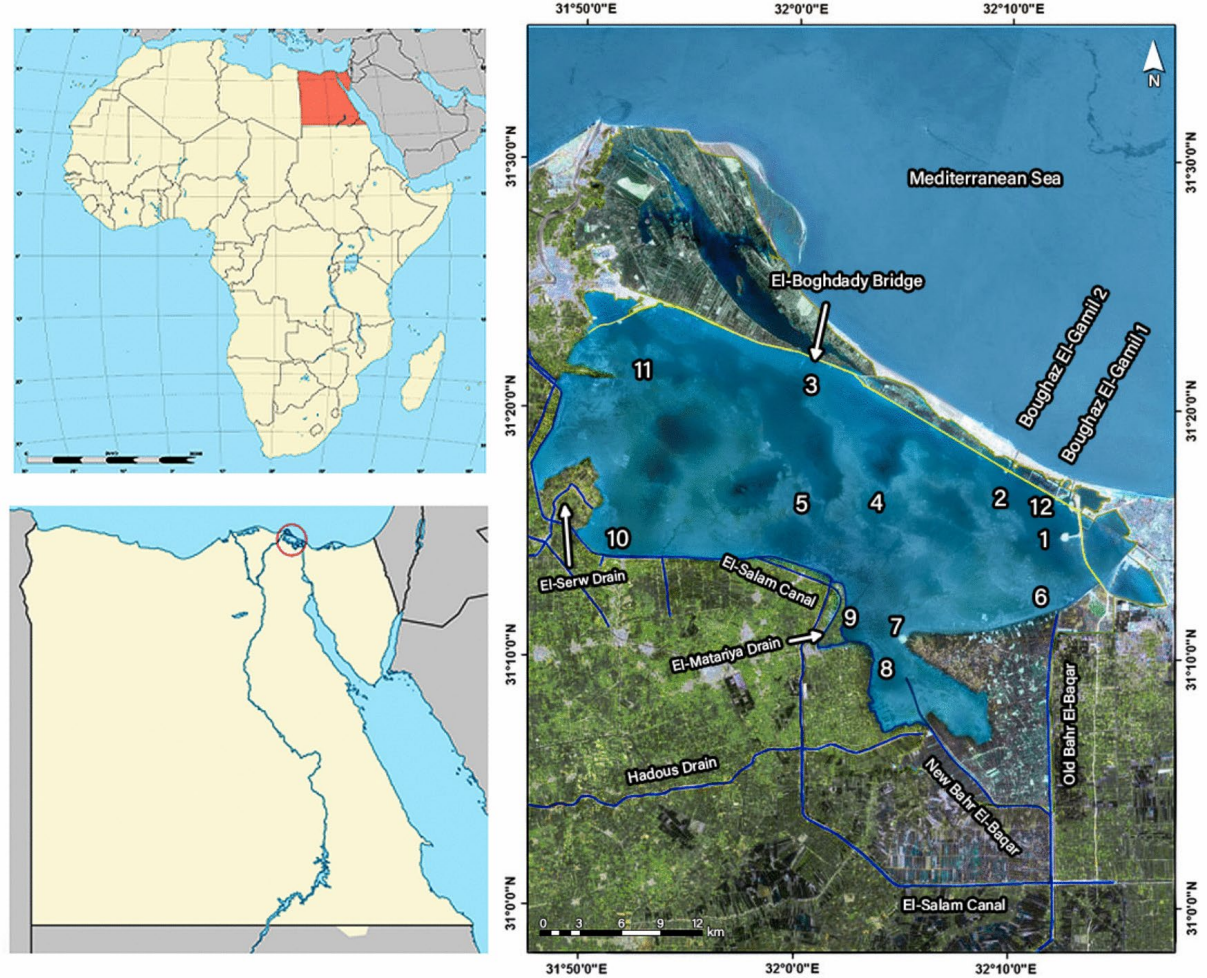
Map of Manzala Lake showing sampling sites after Khedr et al.^12^** (**The map was generated by ArcGis pro 3.1.0, https://experience.arcgis.com/experience/968e183c67774fa786298483a001438d/page/ArcGIS-License-request//).

Samples were collected during the winter and summer seasons of 2015 and 2022 from 12 sites, dividing the lake into three sections: north, middle, and south/southeast. The northern section includes sites 1, 2, 3, 11, and 12, and extends along the Mediterranean coast and is connected to it by several canals. The middle section is represented by sites 4 and 5, located at the center of the lake. The southern and southeast sections comprise stations 6, 7, 8, 9, and 10, which are characterized by receiving significant volumes of water discharged from various domestic, agricultural, and industrial sources (Fig. 1 and Table 1).

**Table 1:** The details of the sampling locations. *WWTP* Wastewater treatment plant, *D* Domestic waste, *A* Agriculture drainage.

Site	Feature	Latitude	Longitude
1	Receives seawater via El-Gmail outlet, and affected by discharge of WWTP*	31° 14′ 49.66ʺ N	32° 11′ 58.78ʺ E
2	Receives seawater via Ashtoum El-Gmail (El-Gmail 2) outlet	31° 17′ 17.97ʺ N	32° 09′ 53.49ʺ E
3	Receives seawater via El-Boghdady opening	31° 20′ 47.80ʺ N	31° 59′ 50.94ʺ E
4	In the middle of the lake, far from the pollution sources, and characterized by fishing activates	31° 16′ 4.88ʺ N	32° 03′ 42.61ʺ E
5		31° 16′ 31.33ʺ N	32° 00′ 49.55ʺ E
6	In front the Old Bahr El-Bqar Darin	31° 12′ 02.64ʺ N	32° 12′ 11.01ʺ E
7	Receive different wastes (mainly D and A)* from several drains including new Bahr El-Bqar Darin	31° 11′ 05.18ʺ N	32°04′54.62" E
8		31° 10′ 24.25ʺ N	32° 04′ 28.71ʺ E
9	At El-Mataryia City and close to El-Mataryia Drain	31° 11′ 25.20ʺ N	32° 02′ 21.13ʺ E
10	At El-Serw City and close to El- Serw Drain	31° 15′ 14.21ʺ N	31° 51′ 29.45ʺ E
11	Far from the pollution sources and affected by saline water	31° 22′ 11.01ʺ N	31° 53′ 17.53ʺ E
12	Infront of El-Gmail outlet1	32° 12′ 39.20ʺ	31° 16′ 7.53ʺ

### Sampling program and analyzing technique

Triplicate subsurface water samples were collected using a Ruttner Water Sampler (2L) and transferred into polyvinyl chloride Van Dorn plastic bottles, which were stored in an ice box until analysis. Biological oxygen demand (BOD) samples were collected in glass-stoppered oxygen bottles, which were carefully filled with water samples. The physicochemical parameters, including temperature, electrical conductivity (EC), dissolved oxygen (DO), and pH, were measured in situ using the Multiparameter Hydrolab model (MultiSet 430i WTW, Germany) after calibration.

The transparency (penetration of light) was measured using the Secchi Disk (30 cm diameter). Total dissolved solids (TDS) were carried out using evaporation of known volumes^37^. BOD was carried out using the 5-day method. COD was measured using the potassium permanganate method. Nutrients were measured by colorimetric methods using a double beam UV/visible spectrophotometer (model Jenway 680, United Kingdom) at wave lengths of 543 nm "formation of reddish purple azo-dye for nitrite (NO_2_) and nitrate (NO_3_) after Hg reduction", 640 nm "phenate method for ammonia (NH_4_)", and 880 nm "ascorbic acid molybdate method for orthophosphate (PO_4_)"^37^. Total nitrogen (TN) and total phosphorus (TP) were quantified as NO_3_ and PO_4_, respectively, following persulfate digestion^38^. The analysis of triplicate samples for each site was conducted to ensure the precision of the analytical methods employed.

Zooplankton samples were collected by filtering 50 L of surface water through a plankton net with a mesh size of 20 μm. The samples were then concentrated to a final volume of 50 mL. One milliliter from each of five subsamples was examined using a binocular research microscope at magnifications of 100× and 400×. Zooplankton were identified to the lowest possible taxonomic level (species) using references from the taxonomic keys^39–45^. Zooplankton abundance was calculated according to APHA^37^ and expressed as individuals per cubic meter (ind./m^3^).

### Statistical analysis

The coefficient of variation (CV) percentage was calculated using Microsoft Excel 2019. Discriminant analysis was employed to categorize similar sites based on chemical and zooplankton data using XLSTAT 2016 software. The normality of the examined data was evaluated using the Shapiro–Wilk test (p > 0.05), and homogeneity of variance was assessed with Levene’s test (p > 0.05). One-way ANOVA (α = 0.05), performed using XLSTAT 2016 software, was used to determine differences in water quality parameters among the lake’s sectors (north, middle, and south) in 2015 and 2022. Additionally, one-way ANOVA was conducted to compare different groups of the lake in 2015 and 2022 based on the abundance and number of saline species, freshwater species, total zooplankton, total rotifers, total copepods, total protozoa, and total cladocerans. Principal component analysis (PCA) was performed to illustrate the relationship between the most abundant zooplankton species and the main chemical parameters using XLSTAT 2016 software.

## Results

### Water quality

The results presented in Table 2 indicate that water salinity varied widely, ranging from 1.28 to 22.52‰ in 2015 and from 1.20 to 39.06‰ in 2022. The highest salinity values were recorded in the northern sector during the summer season at sites 3 and 12, respectively. Conversely, the lowest salinity values were found in the southern sector at sites 8 and 9. Similarly, the highest dissolved oxygen (DO) levels, measuring 12.22 mg/L and 12.7 mg/L, were observed in the northern sectors during the summer at sites 3 and 12, while the lowest DO levels of 0.39 mg/L and 0.66 mg/L, were recorded in the southern sector at sites 8 and 6 in 2015 and 2022, respectively. Additionally, nutrient levels, particularly ammonium (NH_4_), total nitrogen (TN), phosphate (PO_4_), and total phosphorus (TP), along with biochemical oxygen demand (BOD) and chemical oxygen demand (COD), exhibited the highest concentrations in the southern sector, whereas the lowest concentrations were observed in the northern and middle sectors.

**Table 2:** Descriptive statistics data of physicochemical characteristics for Manzala Lake water during 2015 and 2022.

Year	2015			2022		
parameter	Range	Mean ± SD	CV	Range	Mean ± SD	CV
Temp. °C	12.4–26.9	19.74 ± 6.56	33.24	12.29–31.52	21.62 ± 8.73	40.38
Trans.cm	10–60	30.42 ± 11.6	38.14	15–60	37.29 ± 15.6	41.84
EC mS cm^−1^	1.83–32.17	9.35 ± 9.24	98.83	1.7–55.08	13.58 ± 14.17	94.18
Salinity ‰	1.28–22.52	6.54 ± 6.47	98.83	1.20–39.06	10.53 ± 9.92	94.18
pH	7.31–8.99	8.29 ± 0.4	4.77	7.46–8.94	8.38 ± 0.45	5.37
DO mg L^−1^	0.39–12.16	5.73 ± 3.17	55.38	0.68–12.47	6.96 ± 3.27	47.34
BOD mg L^−1^	8.16–85.16	29.36 ± 21.28	72.47	5.69–66.12	23.26 ± 18.16	78.06
COD mg L^−1^	15.91–151.76	60.48 ± 45.13	74.62	10.45–134.69	49.12 ± 41.51	84.52
NO_2_–N µg L^−1^	0–219.4	102.32 ± 78.8	77.01	0.00–346.11	87.98 ± 107.77	122.49
NO_3_–N µg L^−1^	103.71–816.14	339.05 ± 188.64	55.64	28.19–644.31	205.91 ± 178.44	86.66
NH_4_–N µg L^−1^	0.16–11.19	3.01 ± 3.54	118.29	0.11–9.23	2.29 ± 3.02	131.82
PO_4_–P µg L^−1^	86.75–649.28	259.83 ± 184.59	71.04	16.3–432.86	148.51 ± 125.47	84.49
TN mg L^−1^	0.44–16.99	4.92 ± 5.13	104.24	0.27–13.09	3.7 ± 4.25	114.81
TP µg L^−1^	148.37–949.96	466.94 ± 277.46	59.42	76.13–918.85	378.35 ± 290.51	76.78

**Fig. 2:**
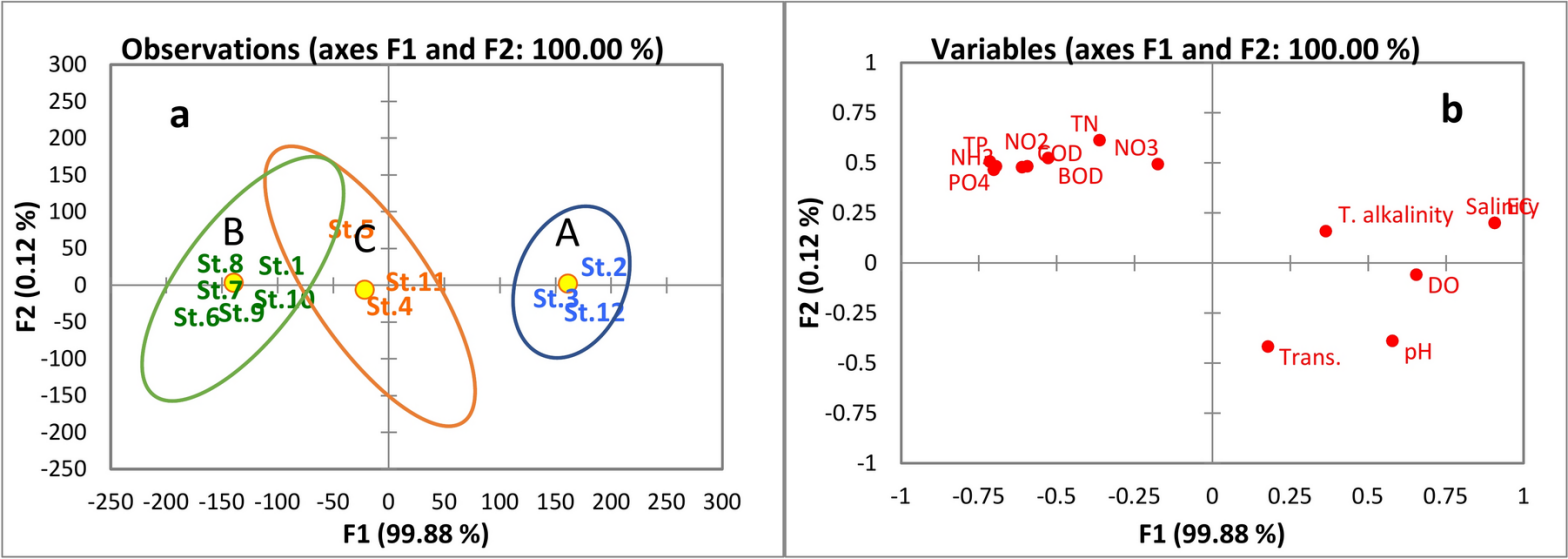
Discriminant analysis of the water quality parameters in Manzala Lake in 2015 (**a**) clustered the lake sites into three groups according to the water quality parameters and (**b**) clustered the water quality parameters based on the high and low values.

**Fig. 3:**
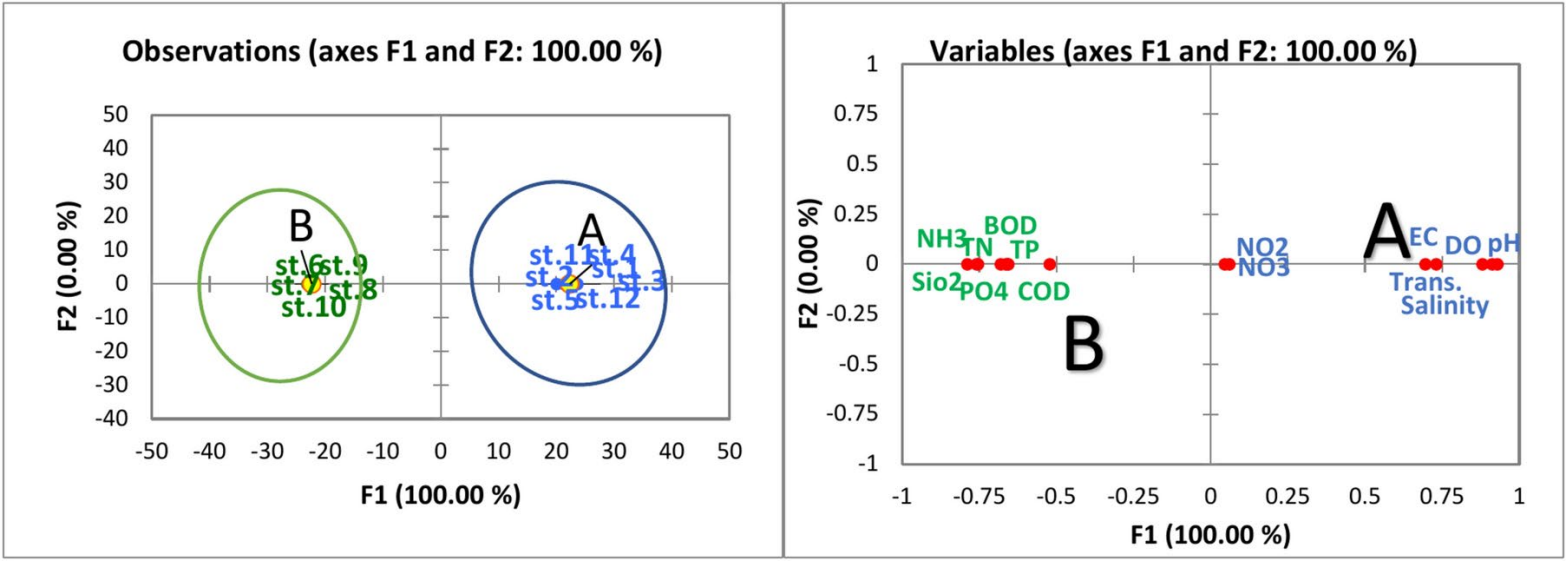
Discriminant analysis of the water quality parameters in Manzala Lake in 2022 (**a**) clustered the lake sites into two groups according to the water quality parameters and (**b**) clustered the water quality parameters based on the high and low values.

Figure 2 illustrates the discriminant analysis based on water quality parameters from 2015. Notably, the sampling sites of the lake were categorized into three groups: A, B, and C. Group A comprised the northern sites (St. 2, 3, and 12) and was characterized by elevated levels of salinity, electrical conductivity (EC), pH, dissolved oxygen (DO), and transparency (Table 3). Groups B and C exhibited some overlap. Group B included the southern sites (St. 6, 7, 8, 9, and 10), along with St. 1 in the northeast. This group was distinguished by high mean values of TN, TP, NH_4_, NO_3_, NO_2_, PO_4_, BOD, and COD. Conversely, Group C consisted of St. 11 (northwest) and St. 4 and St. 5 (located in the middle of the lake). This group was characterized by mean values of all parameters that fell between those of the other two groups. In a similar context, the 2022 discriminant analysis based on water quality parameters classified the various sites of the lake into two groups: A and B. Group A included the northern and middle sites (St. 1, 2, 3, 4, 5, 11, and 12) and was characterized by high mean values of salinity, DO, EC, pH, NO_2_, NO_3_, and transparency. In contrast, Group B encompassed the southern sites (St. 6, 7, 8, 9, and 10) and was characterized by elevated levels of TN, TP, NH4, PO4, BOD, and COD, as depicted in Fig. 3.

**Table 3:** Means and ANOVA data for water quality parameters among the lake site groups in 2015 and 2022.

Parameter	2015			2022			North 2022 vs North 2015		Middle 2022 vs Middle 2015	South 2022 vs South 2015
	North	Middle	South	North	Middle	South	P value			
Transparency cm	31.00 b	43.75 a	24.50 c	40.50 ab	57.50 a	30.50 b	< 0.0001	0.000		0.072
EC mS cm^−1^	17.06 a	5.73 b	3.08 b	23.76 a	18.55 a	4.93 b	< 0.0001	0.001		0.661
Salinity ‰	11.95 a	4.01 b	2.15 b	16.63 a	12.98 a	3.45 b	0.000	0.001		0.661
pH	8.62 a	8.35 ab	7.95 b	8.65 a	8.61 a	8.02 b	0.119	0.589		0.171
DO mg L^−1^	8.13 a	5.89 ab	3.26 b	9.21 a	6.81 ab	4.77 b	0.086	0.097		0.009
BOD mg L^−1^	14.57 b	14.86 b	49.97a	9.74 b	9.99 b	19.85 a	0.000	0.004		0.018
COD mg L^−1^	30.15 b	28.96 b	103.43 a	22.18 b	21.49 b	87.11 a	< 0.0001	0.002		< 0.0001
NO_2_–N µg L^−1^	77.23 b	71.81 b	139.61a	100.41 a	62.69 b	85.68 b	< 0.0001	0.001		0.026
NO_3_–N µg L^−1^	319.53a	261.22 a	389.70 a	209.42 a	212.23a	199.88 a	< 0.0001	< 0.0001		0.180
NH_4_–N µg L^−1^	0.878 b	0.595 b	6.07 a	0.67 ab	0.37 b	4.68 a	0.001	0.657		0.000
PO_4_–P µg L^−1^	137.70 b	155.09ab	423.85a	79.41a	76.16 b	246.53 a	< 0.0001	< 0.0001		0.003
TN mg L^−1^	1.827 b	1.413 b	9.422 a	1.406 b	0.977 b	7.106 a	0.001	0.330		0.000
TP µg L^−1^	268.07b	298.47 b	733.20a	216.27b	226.61b	601.13 a	< 0.0001	< 0.0001		0.017

Different letters indicate the variation between the different sectors in the same year, and the P value indicates the difference for each sector between 2015 and 2022.

### Zooplankton

**Fig. 4:**
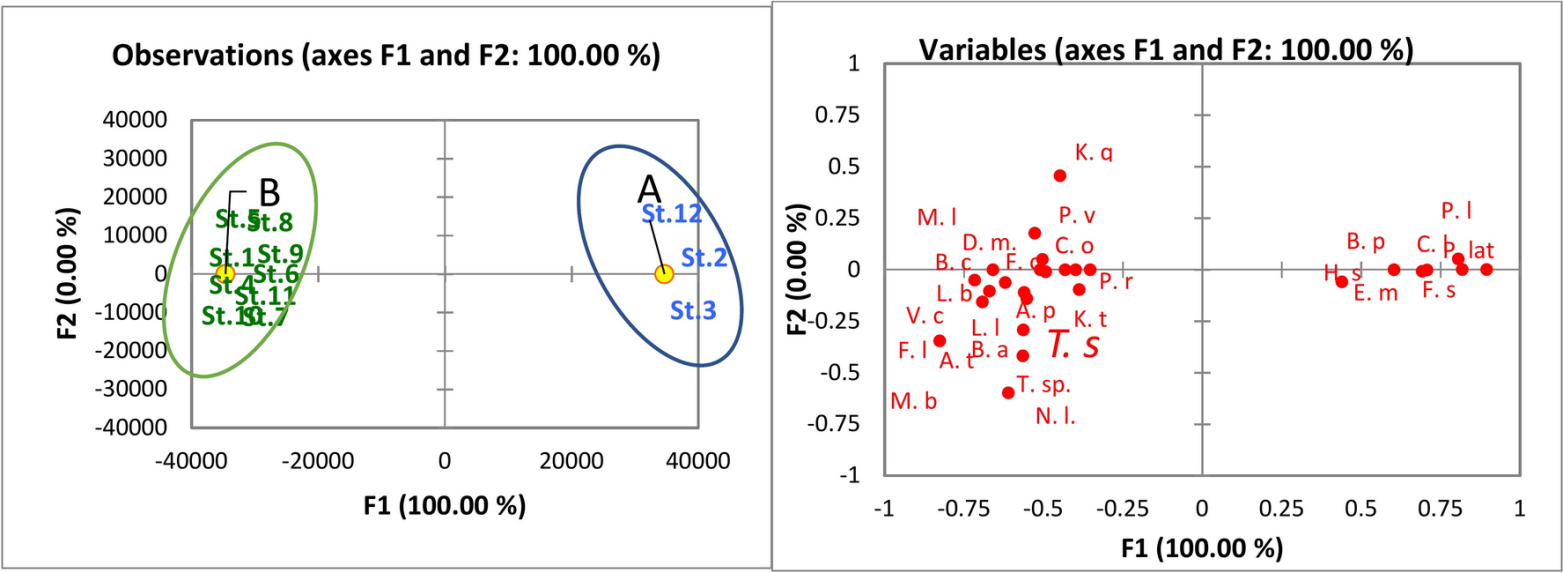
Discriminant analysis of the distribution of zooplankton species in Manzala Lake in 2015, (**a**) clustered the lake sites into two groups according to the distribution of zooplankton species, and (**b**) clustered the dominant zooplankton species into two groups; (*Keratella quadrata* (K. q), *Lecane luna* (L. l), Filinia *opoliensis* (F. o), *Polyarthra vulgaris*, *Centropyxis aculeata* (C.o), *Daphnia magna* (D. m), *Keratella tropica* (K. t), *Brachionus calyciflorus* (B. c), *Lecane bulla* (L. b), Brachionus *angularis* (B. a), *Vorticella campanula* (V. c), *Asplanchna priodonta* (A. p), *Mesocyclops leuckarti* (M. l), *Filinia longiseta* (F. l), *Acanthocycl opstrajani* (A. t), *Moina brachiata* (M. b), Trichocerca stylata *(T. b), Thermocyclops sp. (T. sp.), Nematoda larvae (N. l), Philodena roseola (P. r), Polychaeta larve (P. l),* Brachionus*plicatilis (B.p),* Paracartia latisetosa *(P. lat), Helicostomella subulate (H. s), Euplotes minuta (E. m), Favella serrata (F. s), Cirripidea larvae (C. l)*)*.*

In 2015, a total of 43 zooplankton species were identified, including 30 species of Rotifera, 4 species of Copepoda, 6 species of Protozoa, and 3 species of Cladocera, along with larval forms of Polychaeta, Cirripedia, and Nematoda (Meroplankton). The discriminant analysis of zooplankton species habitats categorized the various sites of the lake into two groups: A and B. Group A was distinguished by the presence and abundance of saline species, which included sites 2, 3, and 12. The saline species were represented by protozoan *Favella serrata* (Möbius, 1887), *Helicostomella subulate* (Ehrenberg, 1833), *Euplotes minuta*, rotifer *Brachionus plicatilis* (Müller, 1786), and copepod *Paracartia latisetosa* (Kričagin, 1873), as well as meroplanktonic larvae of Cirripidae and Polychaetes. *B. plicatilis* were the most abundant species of zooplankton. It formed 22.7% of the total zooplankton count, with the highest density of 976,507 ind./m^3^ at St. 3. Group B was characterized by the presence and abundance of freshwater species in sites 4, 5, 6, 7, 8, 9, 10, and 11 (Fig. 4). Notably, *B. plicatilis* was the only saline-brackish species recorded in group B sites, but it was recorded with low densities at St. 4, 5, 10, and 11. *Brachionus angularis* (Gosse, 1851) and *B. calyciflorus* (Pallas, 1766) were the dominant freshwater rotifers; they contributed 13.4 and 9.9% of total zooplankton abundance, respectively. *B. angularis* attained the highest density (682,500 ind. /m^3^) at St. 6 and 7, while *B. calyciflorus* was more abundant at St. 7 and 9, with average densities of 90,000 and 85,000 ind./ m^3^, respectively. Also, *Filinia longiseta* (Ehrenberg, 1834), *Keratella tropica* (Apstein, 1907), *Philodina roseola* (Ehrenberg, 1832), *Lecane luna* (Müller, 1776), *L. bulla* (Gosse, 1851), and *Polyarthra vulgaris* (Carlin, 1943) were abundant in group B sites. *Acanthocyclops trajani* (Petkovski, 1989) was the most abundant copepod (0.8% of total zooplankton abundance); it was recorded at all sites in Group B. The cladoceran *Moina brachiata* (Jurine, 1820) was the most abundant species; it attained the highest density of 10,500 ind. /m^3^ at St. 4. *Vorticella campanula* (Ehrenberg, 1838) was an abundant protozoan species. It shared 0.3% of the total zooplankton count; it was only recorded at group B. sites.

**Fig. 5:**
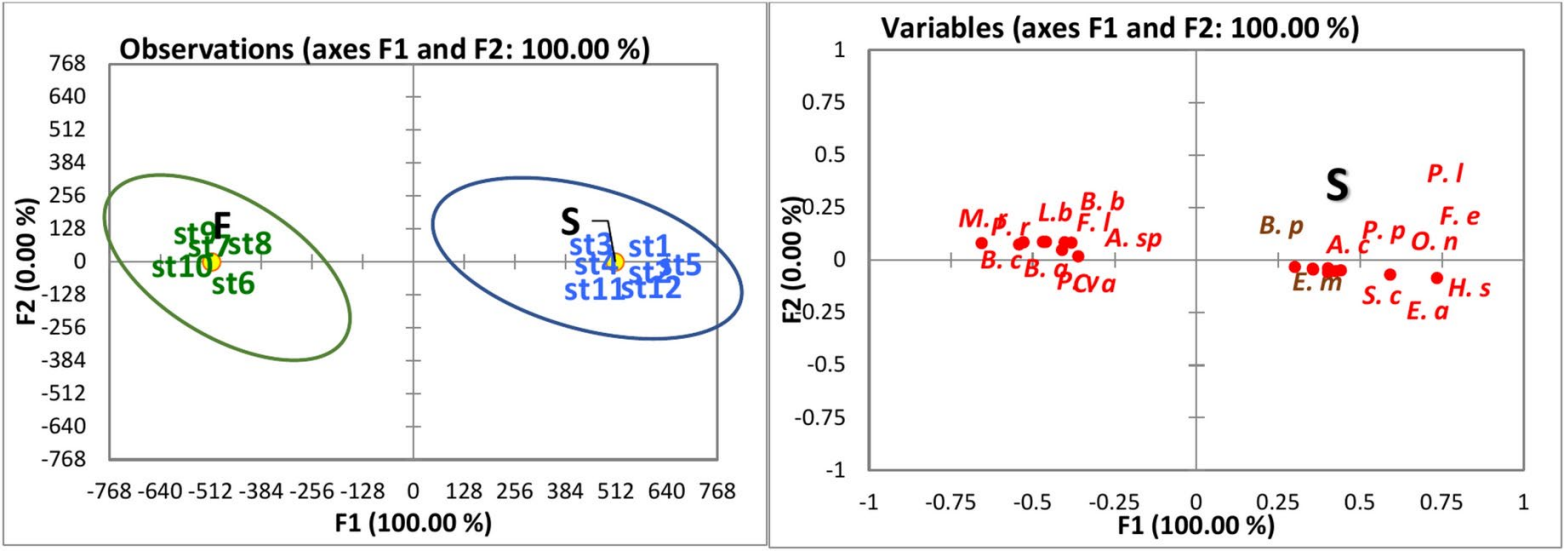
Discriminant analysis of the distribution of zooplankton species in Manzala Lake in 2022 (**a**) clustered the lake sites into two groups according to the distribution of zooplankton species, and (**b**) clustered the dominant zooplankton species into two groups; (Brachionus *budapestinensis* (B.b), *Lecane bulla* (L. b), *Asplanchina sp. (A. sp.), Centropyxis aculeata* (C.o), *Brachionus calyciflorus* (B. c), *Brachionus* angularis (B. a), *Filinia longiseta (F. l), Moina rectirostris (M. r), Polyarthra vulgaris (P. v), Brachionus plicatilis (B.p), Acartia clausii (A. c), Polychaeta larve (P. l), Favella ehrenbergi (F. e), Oithona nana (O. n), Paracalanus parvus (P. p), Helicostomella* subulate *(H. s), Synchaeta calva (S. c), Euterpina acutifrons (E. a).*

In 2022, zooplankton was represented by 31 species, including 16 species of Rotifera, 6 species of Copepoda, 7 species of Protozoa, and 2 species of Cladocera, in addition to the larval forms of Polychaeta, Cirripedia, and Nematoda (Meroplankton). The lake sites were categorized into two groups (A and B) based on discriminant analysis (Fig. 5). Group A comprised the northern and middle sites (1, 2, 3, 4, 5, 11, and 12), while Group B included only the southern sites (6, 7, 8, 9, and 10). The saline protozoan species *Favella ehrenbergii* (Claparède & Lachmann, 1858) and *H. subulate* were only recorded at group A sites. However, the saline protozoan *Euplotes minuta* (Yocum, 1930) was recorded at group A and B sites. It was the dominant protozoan species (3.1% of total zooplankton abundance). The rotifer *B. plicatilis* and *Synchaeta calva* (Lamarck, 1816) were the saline species recorded. *B. plicatilis* was the second most abundant species (25.7%) of total zooplankton abundance, and it was recorded at all sites, but it attained the highest density of 1752 ind/m^3^ at site 11. The other 14 rotifer species were freshwater, and they were constricted to group B sites. Among 11 freshwater rotifers, *B. calyciflorus* and *Brachionus angularis* (Gosse, 1851) were dominant (27.4 and 21.9% of the total zooplankton count, respectively). They were recorded at sites 6, 7, 8, 9, and 10. *Oithona nana* (Giesbrecht, 1892), *Acartia clausii* (Giesbrecht, 1889), *Paracalanus parvus* (Claus, 1863), and *Euterpina acutifrons* (Dana, 1847) were the saline species of Copepod. They were only recorded at group A sites. The saline copepod *Paracartia latisetosa* (Kričagin, 1873) was recorded at sites 1 and 10. Additionally, the freshwater copepod *Acanthocyclops trajani* (Petkovski, 1989) was recorded at sites 1, 4, 6, 7, and 9. *Oithona nana* was the most abundant copepod species (1.3% of total zooplankton abundance). It was observed at sites 2, 3, 4, 10, and 11. *Moina rectirostris* (Leydig, 1860) and *Daphnia longispina* (O.F. Müller, 1776) were the only cladoceran species; they were recorded at sites 7 and 8.

An analysis of variance (ANOVA) was conducted to compare groups A and B in 2015 and 2022, focusing on the abundance and number of saline species, freshwater species, total zooplankton, total rotifers, total Copepoda, total protozoa, and total Cladocera. The comparison between winter and summer was also evaluated based on the aforementioned criteria. Each criterion showed significant differences (P < 0.05) between the two group sites (A and B) in 2015, except for the number of saline species, copepod diversity, cladoceran abundance, and diversity (Table 4). In 2022, the variance between the two group sites was significant across all comparison criteria, except for protozoan diversity and cladoceran diversity. Furthermore, the two years exhibited significant differences across all criteria, except for the abundance and diversity of Rotifera and the abundance of Cladocera.

**Table 4:** The variance analysis (ANOVA) between the sites of group A and group B in each year, as well as the two years according to several zooplankton criteria.

Comparison criteria	Group AVs Group B in 2015		Group AVs Group B in 2022		2015 vs 2022	
	P value	Significant	P value	Significant	P value	Significant
Saline spp. abundance	0.000	Yes	0.000	Yes	0.000	Yes
Number of saline spp.	0.070	No	0.001	Yes	0.000	Yes
Freshwater spp. abundance	0.000	Yes	0.000	Yes	0.000	Yes
Number of Freshwater spp.	0.000	Yes	0.000	Yes	0.036	Yes
T. zooplankton abundance	0.000	Yes	0.000	Yes	0.000	Yes
T. number of zooplankton spp	0.000	Yes	0.008	Yes	0.008	Yes
T. Rotifera abundance	0.000	Yes	0.000	Yes	0.313	No
T. number of Rotifer spp	0.001	Yes	0.000	Yes	0.070	No
T. Protozoa abundance	0.000	Yes	0.000	Yes	0.000	Yes
T. number of Protozoan spp.	0.002	Yes	0.492	No	1.000	No
T. Copepoda abundance	0.000	Yes	0.000	Yes	0.000	Yes
T. number Copepod spp	1.000	No	0.002	Yes	0.005	Yes
T. Cladocera abundance	0.337	No	0.000	Yes	0.588	No
T. number of Cladoceran spp	1.000	No	0.158	No	0.643	No

### The relationship of water quality parameters and zooplankton species

**Fig. 6:**
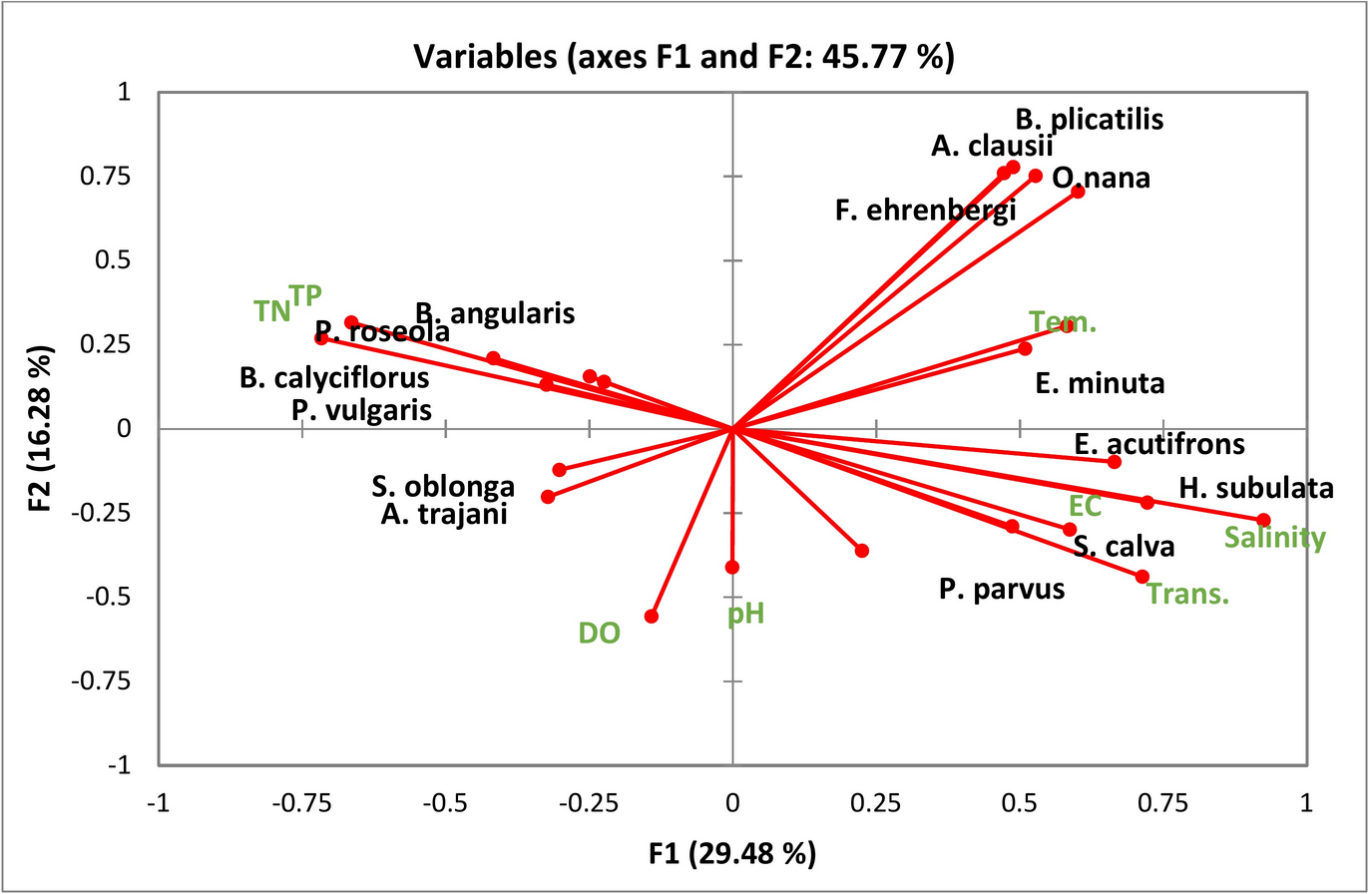
Principal component analysis (PCA) biplot of water quality parameters and the dominant zooplankton species in Lake Manzala in 2015 and 2022.

The PCA analysis (Fig. 6) indicated that salinity was the most influential parameter for zooplankton species, effectively categorizing them into two distinct groups. It exhibited a positive correlation with *F. ehrenbergi*, *H. subulate*, *E. minuta, B. plicatilis* and *S. calva, O. nana*, *A. clausii*, *P. parvus*, and *E. acutifrons.* while demonstrating a negative correlation with other species. Additionally, electrical conductivity (EC) and transparency were positively correlated with saline species. TN and TP showed significant positive correlations with *B. calyciflorus, B. angularis, P. roseola, P. vulgaris, and A. trajani.*

## Discussion

### Water quality

**Fig.7:**
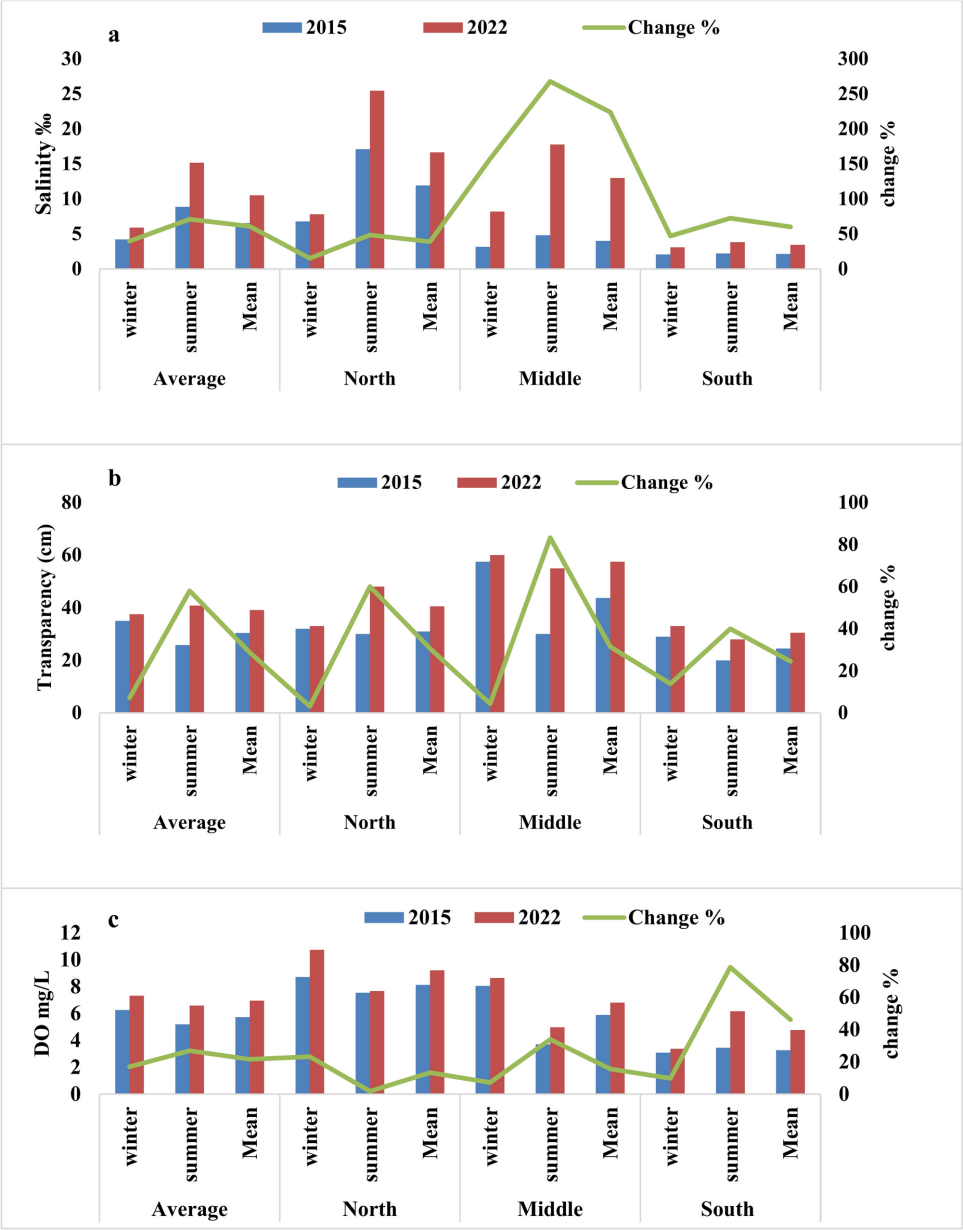
The changes in Salinity (**a**), transparency (**b**), and dissolved Oxygen (**c**) of Lake Manzala water at the different sectors during winter, summer, and mean in 2015 and 2022.

**Fig. 8:**
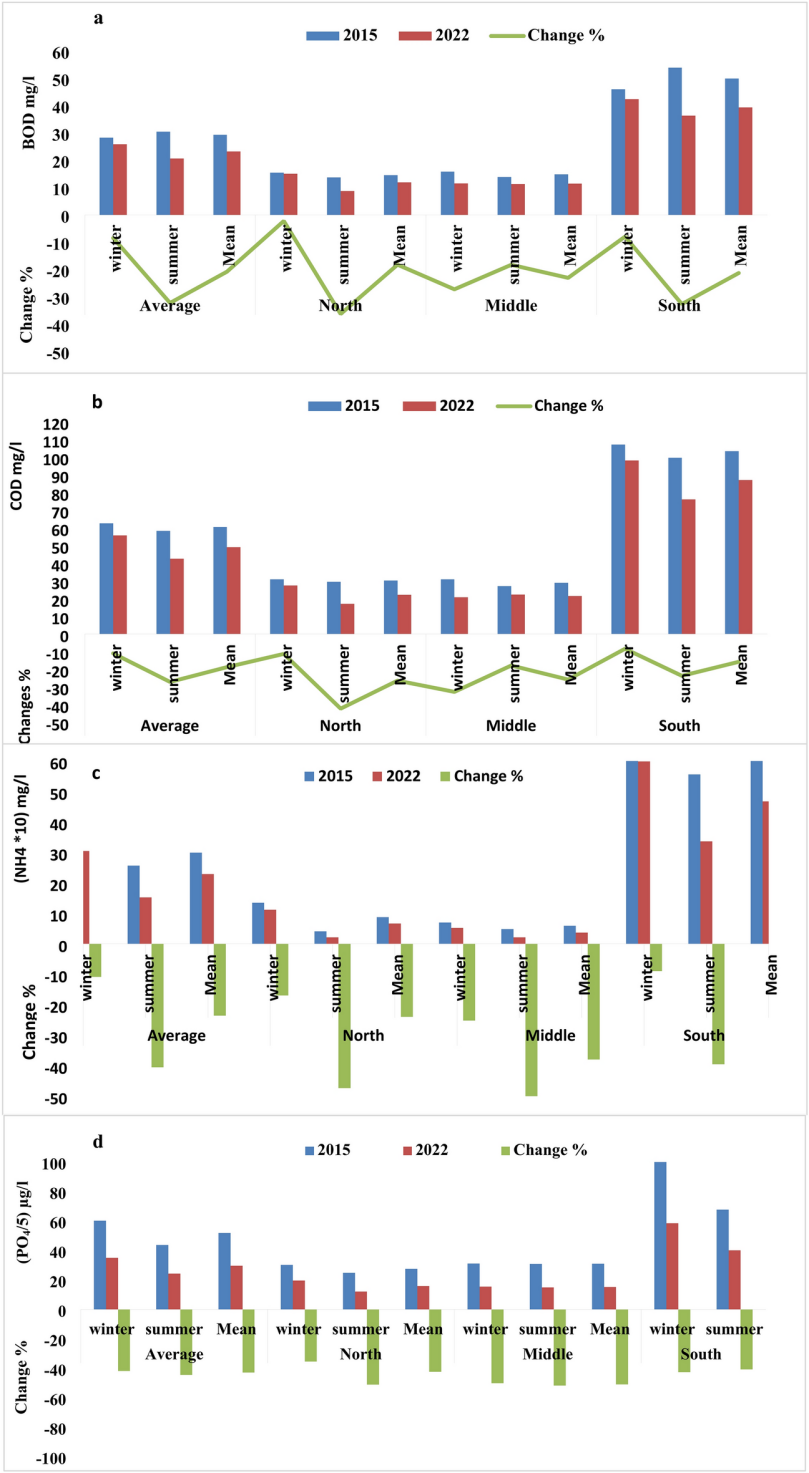
The changes in BOD (**a**), COD (**b**), NH4 (**c**), and PO4 (**d**) of Lake Manzala water at the different sectors during winter, summer, and mean in 2015 and 2022. Values of PO_4_ (ˬµg/L) were divided by 5, and NH_4_ was multiplied by 10 to improve and illustrate the graph.

Dredging operations are among the most prevalent methods for rehabilitating water bodies. Although the final benefits of dredging can vary significantly, it remains one of the most effective solutions for improving water quality by removing pollutants that have accumulated in the sediments of lakes, rivers, streams, canals, ponds, and other bodies of water^21^. In the past decade, Egypt has developed a national strategy aimed at rehabilitating the northern lakes and restoring their natural state. A key component of this strategy includes the dredging and cleansing of lakes, with Lake Manzala being one of the most significant projects. The objectives of the dredging operations extend beyond merely increasing water depth; they also focus on eliminating contaminants from the sediments. Notably, one of the primary goals, particularly in areas adjacent to the Mediterranean Sea, is to facilitate the influx of large volumes of high-quality seawater while simultaneously reducing the amount of wastewater discharged into the lake. This study was conducted in 2015 (prior to dredging and cleansing) and again in 2022 (at the conclusion of the dredging), highlighting the changes in the physicochemical properties of the water that occurred during this period. Regarding our findings, we observed significant increases in the values of water parameters such as transparency, EC, salinity, and DO that indicate an improvement in the water quality, as illustrated in Fig. 7. Conversely, we noted reductions in pollution indicator parameters, such as BOD and COD (Fig. 8), along with decreases in nutrient levels, including ammonia, phosphate, total nitrogen, and total phosphorus.

The highest values of salinity in 2015 (22.50 ‰) and 2022 (38.06 ‰) were recorded in sites close to the sea outlets at St. 3 and St. 12, respectively. Furthermore, the mean salinity levels increased from 4.23, 8.86, and 6.54 ‰ to 5.91, 15.16, and 10.53 ‰, reflecting percentage increases of 39.67%, 71.14%, and 60.96% during winter, summer, and the annual average, respectively. It is noteworthy that water salinity rose to varying degrees across different sectors of the lake. The annual average salinity increased by 39.22%, 223.92%, and 60.23% in the northern, middle, and southern sectors, respectively. The significant increase in salinity in the middle sector indicates a greater incursion and spread of seawater into the lake. Additionally, summer exhibited a higher percentage increase in salinity (71.14%) compared to winter (39.67%). Whereas the percentage increase in salinity was up to 48.72, 276.66, and 72.63% and 15.28, 157.43, and 47.12% in the northern, middle, and southern sectors during summer and winter, respectively (Fig. 7a). Also, the present study recorded higher salinity levels than previous measurements of 5.37, 2.53, 1.1–22.5, 1.1–17.3, and 1.39–21.9% recorded in 1985, 2001, 2004, 2017, and 2020, respectively^46–49^. These findings confirm that the dredging processes have successfully facilitated the entry of substantial amounts of seawater deep into the lake and into regions far from the El-Boughazes outlets. According to several prior studies^1,6^, s seawater is of higher quality than lake water, which is influenced by the influx of various wastewater sources. This ultimately led to a noticeable improvement in the quality of lake water.

Water transparency is one of the most important properties affected by dredging operations. Generally, transparency decreases while turbidity increases during the dredging activities and in the short period following their completion^27^^**.**^ However, the results of the current study contradict this observation, as water transparency increased during both the winter and summer seasons, specifically during dredging process and its final stages. The use of modern dredging equipment may have contributed to this outcome by preventing an increase in turbidity and a decrease in transparency. But the most crucial factor in enhancing transparency is the increased influx of seawater, which is clearer and more transparent, and its distribution in varying proportions within the lake.

Our findings indicate the water transparency of Manzal Lake fluctuated between 10–60 cm in 2015 and 25–65 cm in 2022, reflecting the lake’s turbidity. However, following the dreading process in 2022, transparency improved by 7.14%, 58.06%, and 28.78% during the winter, summer, and annual average, respectively. Consistent with the rising trend of salinity, the rate of transparency increase was more pronounced in the summer with recorded improvements of 60%, 83.33%, and 40% in the northern, middle, and southern sectors, respectively, compared to the winter values of 3.13%, 4.35%, and 13.79%. Additionally, the increase in transparency was most significant in the middle (31.43%), northern (30.65%), and southern (24.49%) sectors, confirming the enhanced incursion and spread of seawater into the lake (Fig. 7b).

In the same trend, DO levels increased by 17.02%, 26.87%, and 21.49% during the winter, summer, and annual averages, respectively. However, the southern sector exhibited a significant increase rate of 9.75% and 78.67% compared to the northern sector, which recorded increases of 23.21% and 1.78%, and the middle sector, which showed increases of 7.2% and 33.92% during winter and summer, respectively (Fig. 7c). It is important to note that the southern region recorded low oxygen values during 2015 and 2022, reaching 0.39 and 0.68 mg/L, respectively. These values are significantly lower than the internationally permissible limits for fish and aquatic organisms, which are 5.5 mg/L for freshwater and 8 mg/L for marine water^50^. These results are consistent with previous studies, which attributed the severe depletion of oxygen in the southern part of the lake to the disposal of large quantities of various types of waste.

In contrast to the fluctuating patterns of oxygen concentration in Lake Manzala during the two study periods, BOD and COD, which are indicators of organic pollution, decreased significantly from average values of 29.36 mg/l and 67.09 mg/l in 2015 to 23.26 mg/l and 58.12 mg/l in 2022, representing a reduction rate of − 20.78% and − 8.79%, respectively. Additionally, the percentage changes in BOD and COD during the summer (− 32.22% and − 27.15 mg/l) were greater than those observed in winter (− 8.45% and − 11.0%), as illustrated in Fig. 8a, b. Furthermore, the most significant reductions were observed in the middle region of the lake, followed by the southern and northern areas. Overall, BOD and COD levels in Lake Manzala remain elevated, exceeding international permissible limits, which indicates a deterioration in the lake’s environmental status. Despite some relative improvement following dredging efforts, BOD and COD values continue to surpass the permissible thresholds necessary for the healthy existence of aquatic organisms. This situation is primarily attributed to the inflow of wastewater laden with organic materials, particularly from the Bahr al-Baqar drain in the southern sector. Consistent with the trends observed in BOD and COD, nutrient levels also exhibited a notable decrease in 2022 compared to 2015 and previous studies^3,10,47,48,50^. The decline rates of the concentrations of ammonia (Fig. 8c), nitrate, total nitrogen, reactive phosphate (Fig. 8d), and total phosphorus were recorded as 10.84%, 19.15%, 35.52%, 12.77%, 41.70%, and 18.97% in winter and 40.58%, − 0.23%, 46.75%, 40.89%, 44.43%, and 18.98% in summer, respectively. Nevertheless, nutrient levels remain high, for example, the levels of ammonia exceeded the permissible limits^10^ in many sites, ranging from 0.21 mg/l to 11.19 mg/l in 2015 and from 0.11 mg/l to 9.23 mg/l in 2022.

The increase in water quality scale in Manzal Lake during 2022 compared to 2015 is confirmed by the discriminant analysis data. Figure 2 shows that the selected sites in Manzala Lake are divided into 3 groups: A-northern (2, 3, and 12), B-southern (sites 6–10), in addition to St. 1 in the northeast, and C-middle (sites 4 and 5) and St.11 in the northwest. The water in the northern sites was of the best quality, followed by the middle sites, then the south. However, it is worth noting that the middle group tends towards and joins the southern group, which indicates an increase in the deterioration of its water properties. It is notable that station 1, which is located in the northwest, belongs to Group B, which has the worst water quality, as a result of the impact of wastewater discharged from the Port Said Wastewater Treatment Plant (Port Said WWTP). This is despite the relative proximity of station 1 of the sea outlet, which indicates the entry of small amounts of seawater and its weak effect within the lake in this area. This may be supported by station 11 (in the northwest) belonging to group C (the middle sector stations). While Fig. 3 shows the division of the stations in 2022 into two groups only, the stations in the middle sector (stations 4 and 5) belonged to the northern group, becoming one group that includes stations (1–5, 11, and 12), which greatly illustrates the clear improvement in the characteristics of the lake’s water in general and especially the middle sector due to the dredging processes. From a general environmental perspective, despite the relative improvement in water quality parameters in 2022, Lake Manzala still faces major challenges as a result of high values of pollution indicators such as BOD, COD, and ammonia in addition to TN and TP. This indicates beyond doubt that it is necessary to reduce the quantity of wastewater and its treatment before discharge into the lake.

### Zooplankton

A total of 43 and 31 zooplankton species were identified in 2015 and 2022, respectively. They included Rotifera (30 and 16 species), Copepoda (4 and 6 species), Protozoa (6 and 7 species), and Cladocera (3 and 2 species), in addition to the larval forms of Polychaeta, Cirripidea, and Nematoda (Meroplankton). Among the 43 zooplankton species, *Favella serrata*, *Helicostomella subulate*, *Euplotes minuta, Brachionus plicatilis*, and *Paracartia latisetosa*, as well as the meroplanktonic larvae of Cirripedes and Polychaetaes, were the only saline taxa recorded in 2015. Furthermore, they were restricted to st. 2, 3, and 12. While the number of saline taxa increased to 12 taxa in 2022, *F. ehrenbergi*, *H. subulate*, *E. minuta, B. plicatilis* and *S. calva, O. nana*, *A. clausii*, *P. parvus*, *P. latisetosa*, and *E. acutifrons,* in addition to meroplanktonic larvae of Cirripidaes and Polychaetaes, were the saline indicators identified in 2022. In comparison with the 2015 and previous studies, the saline species were relatively increased, and they extended in the northern and middle parts of the lake^51^. recorded two adult saline species in the lake: *B. plicatilis*and *P. latisetosa,* as well as Polychaete larvae and Cirriped nauplius. While *P. latisetosa*and *Cirriped* nauplius were the only saline forms recorded by McLaren^52^. Khalifa and Mageed^50^ .recorded *B. plicatilis* and *O. nana,* as well as Cirriped nauplius. Also, Mageed^53^ listed *B. plicatilis, P. parvus, O. nana, Nitocra lacustris*, Polychaete larvae, and Cirriped nauplius as saline forms in Make Manzala. Nevertheless, they were restricted to the northern part of the lake. Abdel Mola and El-Rashid^54^ did not record any saline species in the lake, but this study was conducted in the southern part of the lake. The current study assumed that the increase in the saline species number, especially in the northern and middle parts, may be related to the rise in salinity due to the dredging process. Thus, the lake sites were also grouped into two groups (saline and freshwater) according to the discriminant analysis. Furthermore, the variance between the two groups’ sites was significant according to most comparison criteria based on the ANOVA test. In addition, the PCA analysis showed that salinity was the most effective parameter for zooplankton species; it divided the species into two groups (saline and freshwater). It was positively correlated with *F. ehrenbergi*, *H. subulate*, *E. minuta, B. plicatilis* and *S. calva, O. nana*, *A. clausii*, *P. parvus*, and *E. acutifrons.* Among the saline species*, B. plicatilis* was frequently recorded in all sites, but it was more abundant in high-salinity sites. *B. plicatilis* can live in a wide range of salinity. Therefore, it was the dominant rotifer species in the Egyptian Mediterranean brackish lakes, such as El-Manzala and El-Burullus Lakes^5,54^. It was also the dominant species in saline lakes such as Qarun and Magic Lakes, Egypt^55,56^**.** However, a salinity of up to 20% is the optimum for its growth and reproduction^5^**.**

On the other hand, the freshwater species, mainly rotifers, were more abundant in the southern sites, while they decreased or disappeared in the north and middle parts in 2022. It may be associated with the high eutrophication in the south, which may decrease in the north and the middle part due to the dredging. According to Abde-Aziz and Aboul-Ezz^57^ and Zakaria et al.^58^, freshwater rotifers in the northern lakes in Egypt have increased markedly as a result of eutrophication. Thus, TN and TP were significantly correlated positively with *B. calyciflorus, B. angularis, P. roseola, P. vulgaris, and A. trajani,* while these species were negatively correlated with salinity*.* Ramdani et al.^59^ reported that nutrients and salinity have the greatest influence on the annual and spatial composition of zooplankton changes in Lake Manzal*.* Zhang et al.^24^ suggested that the low abundance of *Brachionus budapestinensis*, *B. angularis*, *B. diversicornis*, and *Synchaeta* spp. is associated with the decline of the trophic state in a shallow eutrophic lake by dredging*.* It is known that the high densities of *Brachionus* spp. are biological evidence for high eutrophication^60^**.** In this respect, the flourishing of *B. calyciflorus* in the southern sites in 2015 and 2022 is associated with high eutrophication due to the discharging of several drains^54^**.** Also, the species was recorded in the Rosetta branch of the river Nile as an indicator species for pollution^61^**.** *B. angularis* is cosmopolitan and has a wide distribution in waters that have a high percentage of eutrophication. Its growth is influenced by salinity and temperature^62^.

Finally, the available zooplankton studies in Lake Manzal are scarce; in addition, these studies have some differences in zooplankton composition. The difference in taxa composition from one study to another may be due to differences in the number of samples, methodology, and inclusion or exclusion of coastal species^53^. However, our results regarding zooplankton composition and distribution in 2022 were markedly different from 2015 and a few previous studies, meaning that dredging may have played a role in changing the ecology of the lake by increasing salinity and reducing nutrients in the northern and middle parts of the lake. Therefore, we can assume that dredging is the main factor influencing zooplankton composition and environmental properties in 2022.

## Conclusion

Lake Manzala is the largest Egyptian Mediterranean lake. Unfortunately, the lake has been exposed to many violations and encroachments over the past decades, such as the shrinking of the area and increased pollution due to receiving huge amounts of a variety of sewage. However, the lake rehabilitation project that began in 2017 through dredging operations hopes to improve its general environmental condition. The current study, which compares the lake status in 2015 (before the dredging) and 2022 (after the dredging), aims to clarify the extent to which dredging operations affect the environmental status of the lake through chemical characteristics and zooplankton communities. The study showed a significant improvement in the water quality and environmental features of Lake Manzala, especially in the northern sector and in the middle of the lake, as a result of the increase in depth due to the dredging process, which led to the entry and spread of seawater to deeper areas within the lake. The results showed an increase in values of salinity, transparency, and dissolved oxygen. In contrast, there were reductions in the values of pollution indicator parameters such as BOD and COD, as well as nutrients (ammonia, phosphate, total nitrogen, and total phosphorus). On the other hand, the results for zooplankton revealed that the saline species were relatively increased in 2022 compared with the 2015 and previous studies, and they extended in the northern and middle parts of the lake. Therefore, we can assume that dredging is the main factor influencing zooplankton composition and environmental properties in 2022. However, from a general environmental perspective, despite the relative improvement in water quality parameters in 2022, Lake Manzala still faces major challenges as a result of high values of pollution indicators such as BOD, COD, and ammonia in addition to TN and TP. This indicates beyond doubt that it is necessary to reduce the quantity of wastewater and its treatment before discharge into the lake. Our study focused on the changes between 2015 and 2022 due to the dredging processes; however, more future studies are planned to determine the long-term changes in the impact of dredging on the environmental condition of the lake.

## Data Availability

All data generated or analyzed during this study are included in this published article.
